# Synthesis of Mesoporous TiO_2_-B Nanobelts with Highly Crystalized Walls toward Efficient H_2_ Evolution

**DOI:** 10.3390/nano9070919

**Published:** 2019-06-26

**Authors:** Ping Li, Qing Cao, Dehua Zheng, Abdulmohsen Ali Alshehri, Yousef Gamaan Alghamidi, Khalid Ahmed Alzahrani, Minjun Kim, Jie Hou, Linfei Lai, Yusuke Yamauchi, Yusuke Ide, Yoshio Bando, Jeonghun Kim, Victor Malgras, Jianjian Lin

**Affiliations:** 1 Key Laboratory of Flexible Electronics (KLOFE) and Institute of Advanced Materials (IAM), Jiangsu National Synergetic Innovation Center for Advanced Materials (SICAM), Nanjing Tech University (NanjingTech), 30 South Puzhu Road, Nanjing 211800, China; 2 Key Laboratory of Eco-chemical Engineering, College of Chemistry and Molecular Engineering, Qingdao University of Science and Technology (QUST), Qingdao 266042, China; 3 State Key Laboratory of Fine Chemicals, Dalian University of Technology, Dalian 116024, China; 4 Department of Chemistry, King Abdulaziz University, Jeddah, P.O. Box. 80203, Jeddah 21589, Saudi Arabia; 5 School of Chemical Engineering and Australian Institute for Bioengineering and Nanotechnology (AIBN), The University of Queensland, Brisbane, QLD 4072, Australia; 6 Department of Plant & Environmental New Resources, Kyung Hee University, 1732 Deogyeong-daero, Giheunggu, Yongin-si, Gyeonggi-do 446-701, Korea; 7 International Center for Materials Nanoarchitectonics (WPI-MANA) and International Center for Young Scientists (ICYS), National Institute for Materials Science (NIMS), 1-1 Namiki, Tsukuba, Ibaraki 305-0044, Japan; 8 Institute of Molecular Plus, Tianjin University, No. 11 Building, No. 92 Weijin Road, Nankai District, Tianjin 300072, China; 9 Australian Institute for Innovative Materials (AIIM), University of Wollongong, Squires Way, North Wollongong, NSW 2500, Australia

**Keywords:** mesoporous materials, TiO_2_ photocatalyst, water splitting

## Abstract

Mesoporous TiO_2_ is attracting increasing interest due to properties suiting a broad range of photocatalytic applications. Here we report the facile synthesis of mesoporous crystalline TiO_2_-B nanobelts possessing a surface area as high as 80.9 m^2^ g^−1^ and uniformly-sized pores of 6–8 nm. Firstly, P25 powders are dissolved in NaOH solution under hydrothermal conditions, forming sodium titanate (Na_2_Ti_3_O_7_) intermediate precursor phase. Then, H_2_Ti_3_O_7_ is successfully obtained by ion exchange through acid washing from Na_2_Ti_3_O_7_ via an alkaline hydrothermal treatment. After calcination at 450 °C, the H_2_Ti_3_O_7_ is converted to a TiO_2_-B phase. At 600 °C, another anatase phase coexists with TiO_2_-B, which completely converts into anatase when annealed at 750 °C. Mesoporous TiO_2_-B nanobelts obtained after annealing at 450 °C are uniform with up to a few micrometers in length, 50–120 nm in width, and 5–15 nm in thickness. The resulting mesoporous TiO_2_-B nanobelts exhibit efficient H_2_ evolution capability, which is almost three times that of anatase TiO_2_ nanobelts.

## 1. Introduction

Mesoporous metal oxides are used in a wide range applications such as energy conversion and storage [1,2], catalysis [3,4], gas sensors [5], etc., because of their high specific surface area and mesoporous networks. In particular, mesoporous TiO_2_ with regular and interpenetrated porous networks have been demonstrated as an effective photocatalyst due to its low cost, environmental friendliness, good physical and chemical stability, large surface area, pore volume and tunable porous structure [6,7,8]. Many efforts have been devoted to developing diverse techniques towards the synthesis of mesoporous TiO_2_ [9,10,11,12,13,14,15,16,17,18]. In addition, the nanostructured framework can generally not sustain strong annealing processes, since crystallite growth and atomic diffusion rapidly jeopardize the delicate pore walls. The fabrication of mesoporous crystalline TiO_2_ via a simple method has therefore remained a great challenge.

TiO_2_ mainly has three well-known crystallographic polymorphs, anatase (tetragonal, space group *I*4_1_/*amd*), rutile (tetragonal, space group *P*4_2_/*mnm*), and brookite (orthorhombic, space group *Pbca*). TiO_2_-B (monoclinic, space group *C*2/*m*) in contrast, however, has gained less attention and fewer studies have been reported. TiO_2_-B, a metastable crystal structure (*a* = 1.21787 nm, *b* = 0.37412 nm, *c* = 0.65249 nm, and *β* = 107.054°) was first synthesized by Marchand et al. in 1980 [19]. Until now, the synthesis of various TiO_2_-B nanostructures, including 0D (nanoparticles [20,21]), 1D (nanowires [22,23], nanoribbons [24], nanotubes [25], nanorods [26] and nanobelts [27]), 2D (nanosheets [28,29,30]), and 3D (mesoporous microspheres [31] and mesoporous microflowers [32]) nanomaterials have been realized through different techniques, such as ion-exchange from layered titanate, hydrothermal or solvothermal treatments of titanium precursors, alkaline hydrothermal treatment of TiO_2_, etc. It is well known that the morphology and porosity of TiO_2_-B architectures strongly affect the physical and chemical properties. For instance, TiO_2_-B can be utilized as anode material for high-power lithium ion batteries (LIBs) due to its characteristic pseudocapacitive energy storage mechanism. Huang et al. demonstrated the use of graphene as a current collector to construct hybrid graphene/TiO_2_-B nanostructures to optimize the performance [27]. Such a hybrid mesoporous architecture can realize fast electron transport and acceleration of diffusion of lithium ions. Due to large surface area, nanoparticles have been used for solar cell applications. However, the electron trapping/scattering at the grain boundaries usually causes high charge recombination loss, resulting in lower efficiency in solar cells. Although 1D nanorods or nanotubes have gained attention due to enhanced charge mobility and strong light absorption, they suffer from lower surface area and poor crystallinity. It is, therefore, highly desirable to fabricate highly crystalline TiO_2_-B nanostructures with enhanced surface area for photocatalytic applications.

Herein, we demonstrate the successful design and facile synthesis of a new material, mesoporous crystalline TiO_2_-B nanobelts. The resultant mesoporous TiO_2_-B nanobelts are carefully characterized by XRD, SEM, TEM, and N_2_ adsorption-desorption isotherm. Finally, we investigate the performance of H_2_ evolution and compared with commercial anatase TiO_2_ powder (P25).

## 2. Materials and Methods

**Preparation of anatase TiO_2_ and mesoporous TiO_2_-B nanobelts.** Anatase TiO_2_ and mesoporous TiO_2_-B nanobelts were simply obtained via an alkaline hydrothermal procedure. It is well-known that the concentration and reaction time are key factors for controlling the nanostructures. To obtain our targeted mesoporous materials, we further modified the experimental process reported previously [33]. In detail, 0.5 g of P25 powder (Nippon Aerosil Co., Ltd., Tokyo, Japan) was dispersed in 20 mL of 10 M NaOH solution under magnetic stirring for 30 min at room temperature, before being transferred into a 25 mL Teflon-lined stainless-steel autoclave (Parr Instrument Company, Moline, IL, USA), and heated at 180 °C for 48 h. The product was washed with distilled water and 0.1 M HCl and collected by centrifugation three times, and finally dried at 80 °C overnight, yielding protonated titanate nanobelts (H_2_Ti_3_O_7_). The mesoporous TiO_2_-B nanobelts were obtained after further heat-treating the product at 450 °C for 2 h in air. For comparison, anatase TiO_2_ nanobelts were also prepared by annealing the H_2_Ti_3_O_7_ nanobelts at 600 °C and 750 °C for 2 h in air.

**Characterizations**. The crystalline phases of the as-synthesized products were measured and characterized with an X-ray diffractometer (XRD, SmartLab, Rigaku Corporation, Tokyo, Japan) using Cu Kα radiation. Field-emission scanning electron microscopy (FESEM, JSM-7001F, JEOL Ltd., Tokyo, Japan) and transmission electron microscopy (TEM, JEM-2100F, JEOL Ltd., Tokyo, Japan) were used to examine the morphology of the samples. A nitrogen adsorption apparatus (BELsorp-mini II, MicrotracBEL Corp., Osaka, Japan) was used to determine the Brunauer–Emmett–Teller (BET) surface areas (*S*_BET_). All the samples were degassed at 120 °C overnight before measurement.

**Photocatalytic Test for H_2_ Evolution.** Photocatalytic H_2_ evolution was evaluated *via* a well-known method using Pt as a co-catalyst and methanol as a sacrificial agent [34]. Because the H_2_ evolution from water occurs through two-electron reduction, co-catalysts accumulating excited electrons and sacrificial agents scavenging holes are necessary to promote charge separation. The photocatalytic test of anatase TiO_2_ and mesoporous TiO_2_-B nanobelts for H_2_ evolution was performed under AM 1.5 light irradiation (λ > 300 nm, 100 mW·cm^−2^). In-situ deposition of fine Pt nanoparticles on the catalysts were carried out by adding an appropriate amount H_2_PtCl_6_ solution [34]. 70 mg of the 0.5 wt % Pt-loaded nanobelts were dispersed into aqueous solution (220 mL of H_2_O and 50 mL of CH_3_OH) under magnetic stirring.

## 3. Results

**Figure 1:**
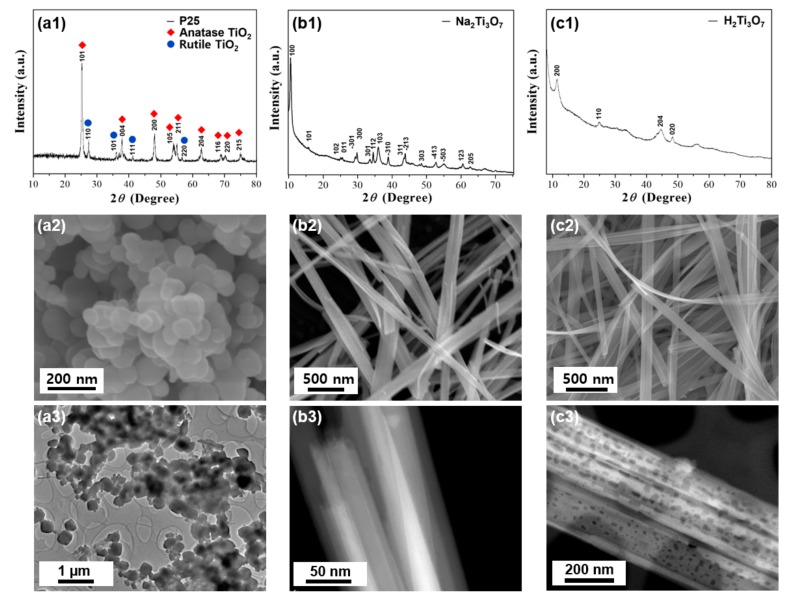
X-ray diffractometer (XRD) patterns, SEM images, and transmission electron microscopy (TEM) images of (**a1-3**) the starting P25 powders, (**b1-3**) Na_2_Ti_3_O_7_ nanobelts, and (**c1-3**) H_2_Ti_3_O_7_ nanobelts, respectively.

In order to understand the formation process, X-ray diffraction (XRD) analysis and SEM observation were carried out to confirm the crystal structure and morphology of the starting P25 powder and the intermediate samples (Figure 1). As mentioned in the experimental section, firstly P25 powders (Figure 1a) were dissolved in NaOH solution under hydrothermal condition, forming an intermediate sodium titanate precursor (Na_2_Ti_3_O_7_) phase, as shown in Figure 1b. In the present study, H_2_Ti_3_O_7_ was obtained by ion exchange through acid washing from Na_2_Ti_3_O_7_ via an alkaline hydrothermal treatment (Figure 1c). The obtained intermediate product is similar to that of a layered H_2_Ti_3_O_7_ (JCPDS 41-0192), featuring a (200) reflection peak at 11°. “Dissolution-Recrystallization” process has been well-known as an important technique for crystal transformation [35]. In the present hydrothermal process, OH^−^ ions diffuse into the P25 powders, causing a gradual dissolution of the TiO_2_ crystals and forming sodium titanate intermediates without pores (Figure 1b3). After ion exchange (HCl solution), H_2_Ti_3_O_7_ nanobelts with pores are obtained (Figure 1c3).

**Figure 2:**
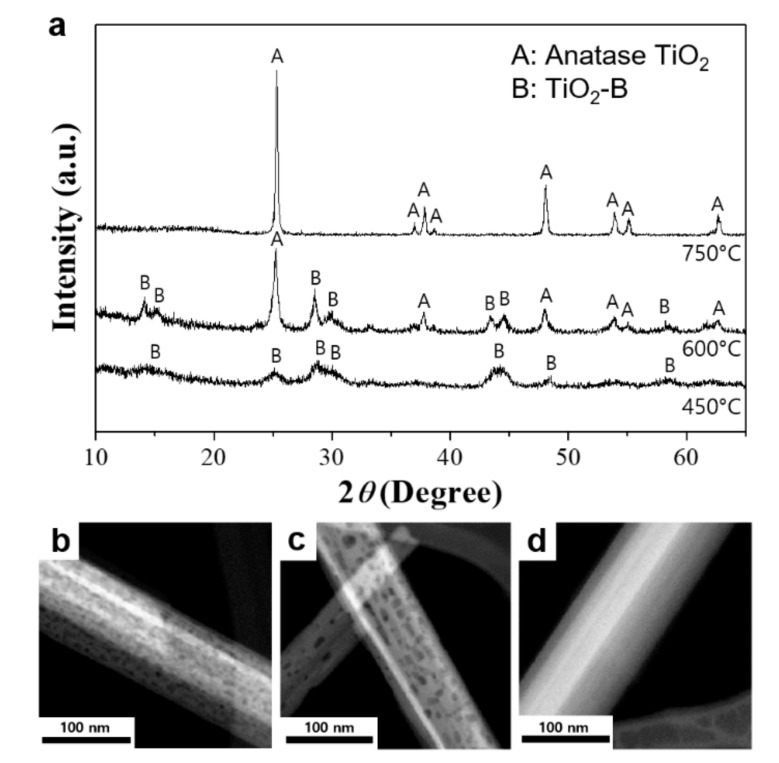
(**a**) XRD patterns of the TiO_2_ nanobelts obtained at different calcination temperatures, TEM images of TiO_2_ nanobelts obtained at (**b**) 450 °C, (**c**) 600 °C, and (**d**) 750 °C.

After calcination at 450 °C, the well-defined peaks located at 2*θ* = 24.9°, 28.5°, 43.5°, 48.1° and 62.9° can be assigned to the (110), (002), (003), (020), (313) diffraction planes of a TiO_2_-B phase, respectively (JCPDS 074-1940, Figure 2a). At 600 °C, the newly formed anatase phase coexists with TiO_2_-B, which completely converts into anatase when annealed at 750 °C. We notice that the porosity in the nanobelt structure is maintained after treating at 600 °C, but is lost when heated beyond 750 °C (Figure 2b–d).

**Figure 3:**
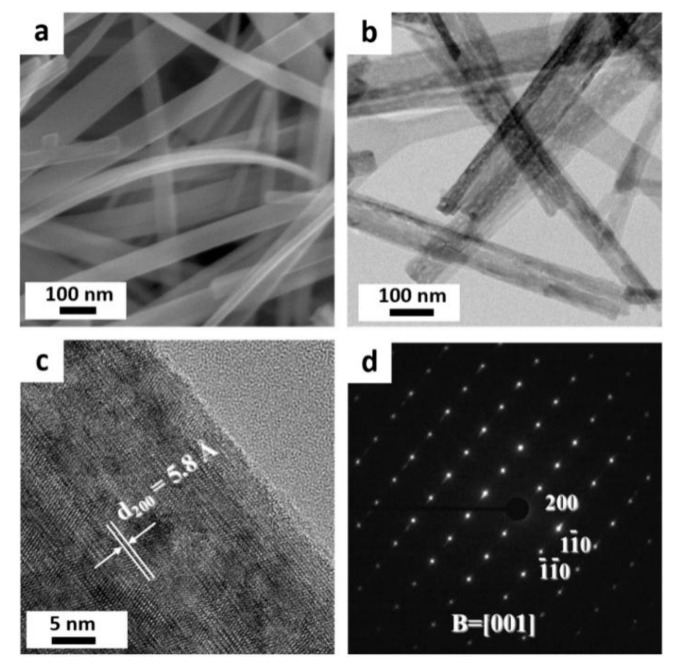
(**a**) Low magnification field-emission scanning electron microscopy (FESEM) and (**b**) TEM image of mesoporous TiO_2_-B nanobelts, (**c**) high resolution TEM (HRTEM) image of one single nanobelt, showing the fringe spacing of crystalline TiO_2_-B and (**d**) the corresponding electron diffraction (ED) patterns.

Figure 3a shows a typical low-magnification SEM image of the mesoporous TiO_2_-B nanobelts annealed at 450 °C. The nanobelts are uniform with up to a few micrometers in length, 50–120 nm in width, and 5–15 nm in thickness, as supported by the low-magnification TEM image (Figure 3b). Interestingly, it can also be seen from the TEM image that the surface of the nanobelts is nanoporous. The high resolution TEM (HRTEM) image (Figure 3c) shows a single mesoporous TiO_2_-B nanobelt with a fringe spacing of ~5.8 Å, which corresponds to the (200) interplanar distance of the TiO_2_-B phase. The clear lattice fringes indicate that this material is highly crystalline in nature. The electron diffraction (ED) pattern in Figure 3d exhibits diffraction spots corresponding to the [001] zone axis of the TiO_2_-B phase. The corresponding fast Fourier transform (FFT) confirms the presence of the (200), (1-10), (-1-10) crystallographic planes of the TiO_2_-B monoclinic *C*2/*m* crystal structure.

**Figure 4:**
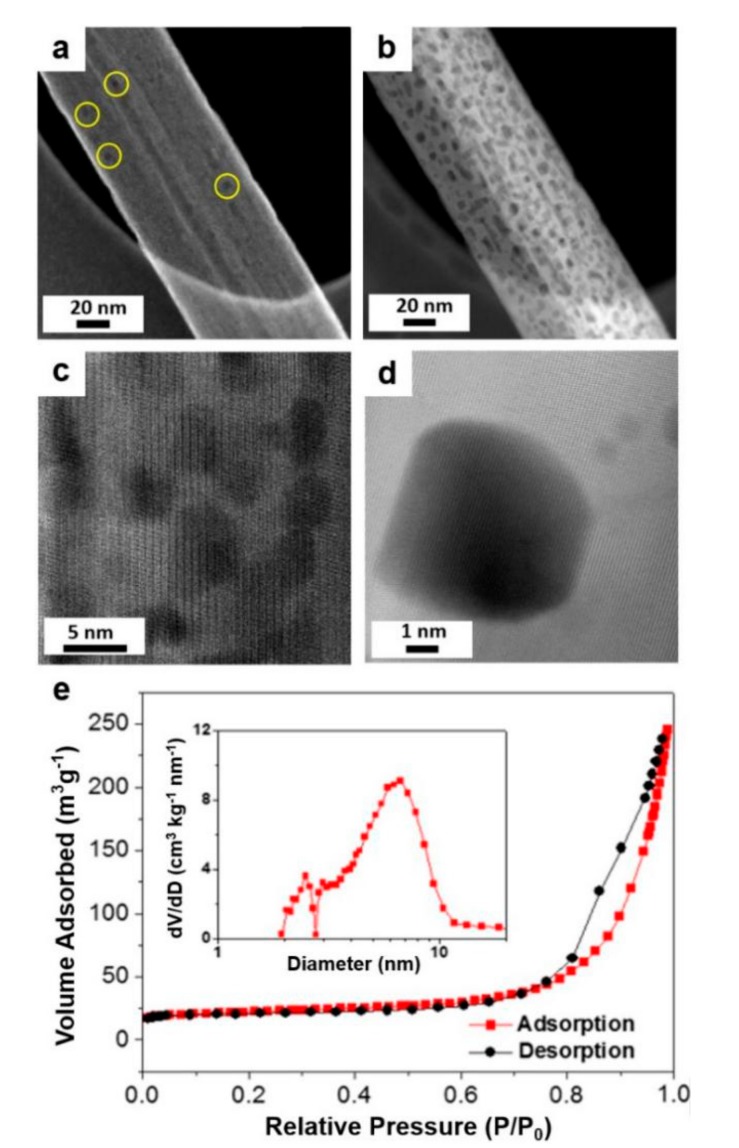
(**a**) High magnification SEM image, highlighting one single TiO_2_-B nanobelt with pores, (**b**) TEM image of mesoporous TiO_2_-B nanobelts, revealing homogeneous nanopores, (**c**) high-angle annular dark-field scanning transmission electron microscope (HAADF-STEM) image of single mesoporous TiO_2_-B nanobelt, (**d**) magnified pore image in (c), and (**e**) N_2_ adsorption-desorption isotherm of the mesoporous TiO_2_-B nanobelts. Inset: the corresponding pore size distribution calculated by the Barrett-Joyner-Halenda (BJH) method from the adsorption curve.

The morphology and porosity of the mesoporous TiO_2_-B nanobelts were further confirmed by electron microscopy characterization. The high magnification SEM image in Figure 4a highlights a single nanobelt, revealing a relatively rough surface with numerous pores, further supported by high-angle annular dark-field scanning transmission electron microscope (HAADF-STEM) (Figure 4b).

The HRTEM image (Figure 4c) shows a clear mesoporous structure, where the high contrast regions correspond to 6–8 nm pores (Figure 4d) uniformly dispersed on the surface. This is also confirmed through the Barrett-Joyner-Halenda (BJH) pore size analysis calculated from the nitrogen (N_2_) adsorption/desorption isotherms (Figure 4e). The isotherms show typical type IV curves with a sharp capillary condensation step at *P*/*P*_0_ = 0.8–0.9. At relatively high pressure, the curves exhibit a small hysteresis. The Brunauer-Emmett-Teller (BET) specific surface area is measured to be 80.9 m^2^ g^−1^.

**Figure 5:**
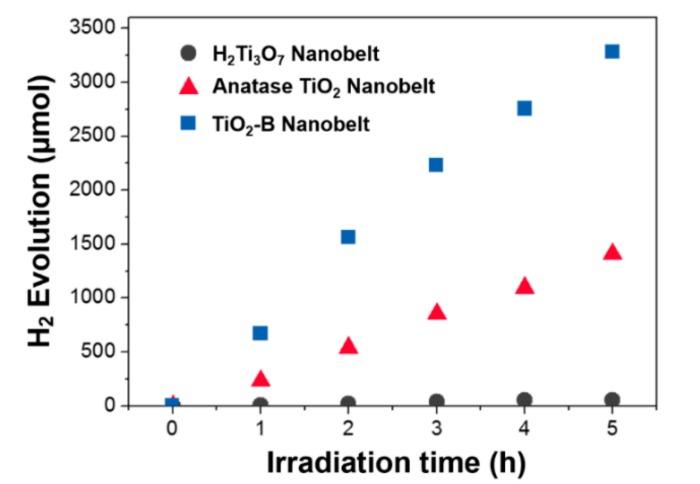
Comparison of photocatalytic water splitting tests of Pt-loaded (0.5%) H_2_Ti_3_O_7_, anatase TiO_2_ and mesoporous TiO_2_-B nanobelts samples with the same mass (70 mg) under UV-Visible light irradiation (λ > 300 nm).

TiO_2_ materials are promising candidates for photocatalysis [36], such as efficient water splitting into H_2_. This half-reaction of water splitting converts solar energy into chemical or electrical energy in an economical way. To evaluate the photocatalytic activities of the anatase TiO_2_ and mesoporous TiO_2_-B nanobelts, H_2_ evolution tests were carried out in the presence of methanol. Figure 5 compares photocatalytic water splitting tests of the mesoporous TiO_2_-B and anatase TiO_2_ nanobelts, both Pt-loaded (0.5 wt%), under the same conditions, i.e., with the same surface area (ca. 0.23 m^2^) and the same mass (70 mg).

As shown in Figure 5, mesoporous the TiO_2_-B nanobelts exhibit a superior photocatalytic activity with a H_2_ evolution rate of 656.10 µmol h^−1^, about three times that of anatase TiO_2_ nanobelts (282.06 µmol h^−1^). Commercially available P25 powders consisting of anatase and rutile crystalline phases cannot realize efficient H_2_ evolution, but after loading with Pt, the H_2_ evolution rate can be greatly enhanced up to 100 μmol h^−1^ (in case of 15 mg catalyst) [37]. Compared to anatase and rutile crystalline phases, TiO_2_-B phase is known to be more active towards H_2_ evolution. Cai et al. reported that pure TiO_2_-B nanobelts without Pt nanoparticles showed a H_2_ evolution rate of ~107 μmol h^−1^ [38]. In our study, the photocatalytic rate of our Pt-loaded mesoporous TiO_2_-B nanobelts greatly increases around 6 times higher than that of pure mesoporous TiO_2_-B nanobelts without Pt [38]. 

## 4. Conclusions

We have designed and fabricated for the first time highly crystallized mesoporous TiO_2_-B with high surface area (80.9 m^2^ g^−1^) and pore size of 6–8 nm. The obtained highly crystallized mesoporous TiO_2_-B was used as photocatalyst, showing efficient water splitting for H_2_ evolution (656.10 µmol h^−1^), almost three times that of anatase TiO_2_ nanobelts (282.06 µmol h^−1^). It opens a new strategy for the design for photocatalyst of H_2_ evolution reaction from H_2_O.
